# A spatial clustering-based approach to design monitoring networks of infectious diseases: a case study of hand, foot, and mouth disease

**DOI:** 10.1186/s40249-025-01331-7

**Published:** 2025-07-28

**Authors:** Shuting Li, Yuanhua Liu, Ke Li, Zengliang Wang, Michael P. Ward, Wei Tu, Jiayao Xu, Rui Yuan, Lele Zhang, Na Wang, Jidan Zhang, Yu Zhao, Henry S. Lynn, Zhaorui Chang, Zhijie Zhang

**Affiliations:** 1 Department of Epidemiology and Health Statistics, Fudan University, Shanghai, China; 2 Shanghai Institute of Infectious Disease and Biosecurity, Fudan University, Shanghai, China; 3 Department of Epidemiology, School of Public Health, Cheeloo College of Medicine, Shandong University, Jinan, Shandong China; 4 Sydney School of Veterinary Science, The University of Sydney, Camden, NSW Australia; 5 Department of Geology and Geography, Georgia Southern University, Statesboro, GA 30460 USA; 6 Division of Infectious Disease, Key Laboratory of Surveillance and Early-Warning On Infectious Disease, National Key Laboratory of Intelligent Tracking and Forcasting for Infectious Disease, Chinese Center for Disease Control and Prevention, 155 Changbai Rd, Changping District, Beijing, China

**Keywords:** Spatial cluster stratified sampling, Monitoring network design, Hand, Foot, and mouth disease, Spatial data analysis, Spatial epidemiology

## Abstract

**Background:**

Effective monitoring of infectious diseases is crucial for safeguarding public health. Compared to comprehensive nationwide surveillance, selecting representative sample cities to constitute the monitoring network for surveillance provides similar effectiveness at a lower cost. We developed Spatial Cluster Stratified Sampling (SCSS) to select sample cities for infectious diseases exhibiting spatial autocorrelation.

**Methods:**

To improve monitoring efficiency for hand, foot, and mouth disease (HFMD), we used SCSS to design a monitoring network, which involved four main steps. First, we used Spatial Kluster Analysis by Tree Edge Removal (SKATER) to stratify the data. Second, we applied the cost–benefit balance to determine the optimal sample size. Third, we performed simple random sampling within each stratum to establish an initial monitoring network. Fourth, we used cyclic optimization to finalize the monitoring network. We evaluated the spatiotemporal representativeness using root mean square error (RMSE), Spearman's rank correlation, global Moran’s *I*, local Getis-Ord G*, and Joinpoint Regression. We also compared the effectiveness of SCSS with *K*-means, traditional stratified sampling, and simple random sampling using RMSE.

**Results:**

The optimal sample size was determined to be 103. Overall, the predicted values for each city significantly correlated with the true values (*r* = 0.81,* P* < 0.001). Both the predicted and true values showed positive spatial autocorrelation (Moran’s *I* > 0,* P* < 0.05), and the sensitivity, specificity, and accuracy of the predicted local Getis-Ord G* values, evaluated against the true values as the gold standard, were 0.76, 0.91, and 0.87, respectively. The weekly predicted values for each city showed significant correlation with the true values (*P* < 0.05). The 95% confidence intervals (*CI*) for the predicted values of joinpoint locations, annual percent change (APC), and average annual percent change (AAPC) encompassed the true values, and the number of joinpoints matched the true values. Among the four methods compared, SCSS exhibited the lowest and most centralized RMSE.

**Conclusions:**

SCSS proved to be more accurate and stable than traditional methods, which overlook spatial information. This method offers a valuable reference for future design of monitoring networks for infectious diseases exhibiting spatial autocorrelation, enabling more efficient and cost-effective surveillance.

**Graphical Abstract:**

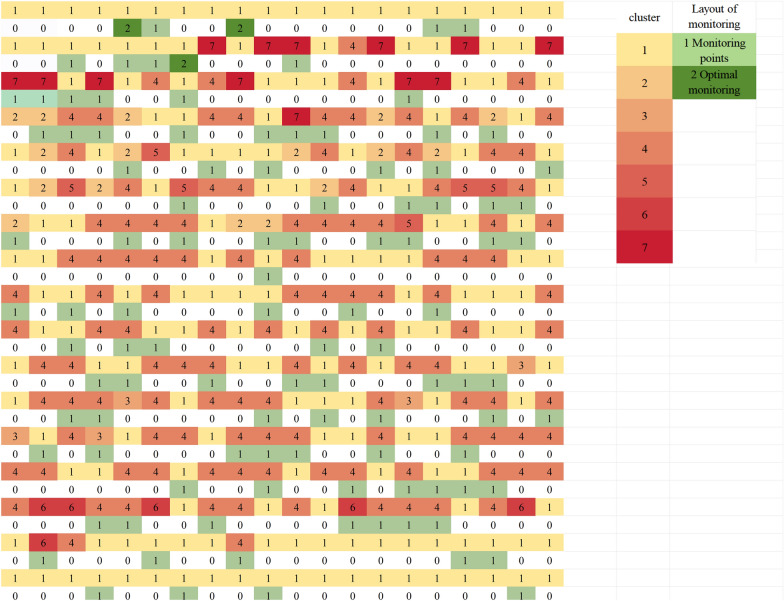

**Supplementary Information:**

The online version contains supplementary material available at 10.1186/s40249-025-01331-7.

## Background

Infectious diseases − such as severe acute respiratory syndrome, influenza A (H1N1) virus infection, Ebola, Zika, Middle East respiratory syndrome, Nipah, and the coronavirus disease 2019 pandemic − have posed a major threat to public health, mental well-being, and socio-economic stability [1]. Effective control of serious infectious diseases relies on robust surveillance systems, which play a vital role in providing early warning, assessing disease spread, and supporting the development of targeted interventions to mitigate the impact of epidemics. In China, 41 notifiable infectious diseases must be reported through a reporting system by medical institutions on a national scale, which is guided and reviewed by the Center for Disease Control and Prevention [2, 3]. Non-notifiable infectious diseases are typically studied through sentinel surveillance, sampling surveys, or outbreak investigations [4, 5]. Unlike non-notifiable infectious diseases, notifiable infectious diseases spread rapidly, present severe symptoms, have high mortality rate, and demand prompt diagnosis and treatment in their early stages. Advances in medical technology and widespread vaccine administration have significantly decreased the severity of some notifiable diseases. For example, most hand, foot, and mouth disease (HFMD) patients in China typically experience mild symptoms, and the number of deaths has remained below nine per year since 2020 [6]. For these self-limiting diseases with mild symptoms, it may be more economically feasible to select representative sample cities for monitoring, from which the national epidemic situation can be effectively inferred. Comprehensive nationwide surveillance is costly and requires significant manpower, resources, and interdepartmental coordination. This broad scope and limited resources present significant challenges to data quality, particularly in terms of completeness, accuracy, consistency, and timeliness [7–9]. Therefore, choosing representative sample cities for monitoring is a more effective strategy. In countries such as Cambodia, Malaysia, Vietnam, and the Republic of Korea, HFMD is not a notifiable infectious disease, and partial regional surveillance is used instead [10, 11]. However, the surveillance database of HFMD in China, where it is classified as a notifiable infectious disease, provides a rare opportunity to study the design of a monitoring network. This is because we have access to the overall situation of HFMD, and the selected representative cities are treated as samples. Their efficiency can be evaluated based on how well they represent the overall situation, allowing for an effective assessment of the quality of the monitoring network formed by the selected representative sample cities.

Previous monitoring network designs were primarily based on expert judgment, covering a fraction of the target population, or traditional stratified random sampling. Expert judgment is subjective and heavily influenced by factors such as the epidemic situation, geographical location, and monitoring conditions. This approach focuses on areas with high incidence, leading to a failure to accurately represent the overall epidemic situation [12]. Determining the sample size based on a specified coverage ratio of the entire population is straightforward and easy to implement. However, it may not provide optimal monitoring efficiency without precise sample size estimation or cost-benefit analysis [13, 14]. Traditional stratified random sampling, which considers factors such as geographic regions, incidence rates, urban and rural areas, economic indicators, and population size as strata [15], often overlooks the spatial distribution of disease and assumes data independence. This assumption has been increasingly questioned due to the first law of geography, which suggests that everything is related to everything else, but nearer things are more closely related than distant things [16]. As a result, spatial imbalance and data redundancy can occur. In contrast, incorporating spatial information (e.g., spatial distribution of disease) into the design of monitoring networks can significantly improve monitoring accuracy and efficiency [17]. The incidence of HFMD at different locations is clearly dependent, and its spatial distribution exhibits certain heterogeneity [18]. Therefore, spatial analysis techniques that integrate spatial information are valuable for setting an HFMD monitoring network.

Using HFMD as an example, this study aimed to establish a methodological framework for designing a cost-effective monitoring network by integrating HFMD spatial information. The study comprehensively evaluated the effectiveness of the monitoring network design from a spatiotemporal perspective, and compared it with traditional methods. The goal was to provide reliable technical and methodological support for the effective design of future infectious disease monitoring networks.

## Methods

### Data sources

Individual case data for HFMD were obtained from China's National Notifiable Infectious Disease Surveillance System, covering both clinically diagnosed and laboratory-confirmed cases in China from January 1, 2018 to December 31, 2019. Case definitions are provided in additional file. Weekly data for individual cases were aggregated across the 340 prefecture-level cities, with complete weekly records available for each city during the entire study period. The number of permanent residents for each prefecture-level city, corresponding to the year of the reported HFMD cases, was obtained from the provincial and municipal Health Statistical Yearbook (https://data.cnki.net/yearBook?type=type&code=A) [20].

The incidence of HFMD (per 100,000) was calculated by using the number of HFMD cases in each city as the numerator and the resident population of the corresponding city as the denominator.

## Statistical analysis

### Design process of the monitoring network

**Fig. 1:**
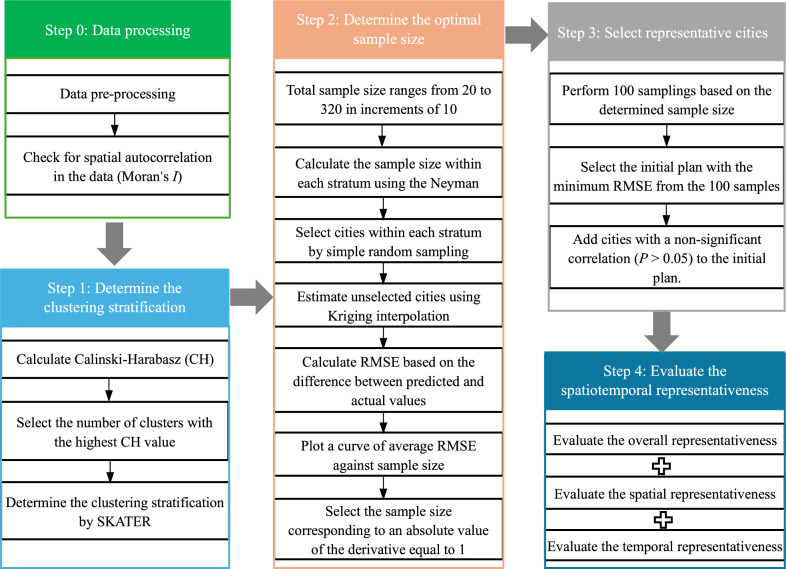
Flowchart of monitoring network design procedures. In the figure, SKATER stands for Spatial Kluster Analysis by Tree Edge Removal

A spatial cluster stratified sampling (SCSS) approach was developed for the design of the monitoring network, which includes four steps: (i) Determine cluster stratification, (ii) Determine the optimal sample size, (iii) Select representative sample cities, and (iv) Evaluating spatiotemporal representativeness (Fig. 1). Using HFMD incidence data from 2018, we selected representative sample cities from 340 cities to form the monitoring network. We then evaluated these cities based on how well they represented the unselected ones. To verify the stability of the representativeness of this monitoring network, we conducted a sensitivity analysis using data from 2019.

All statistical analyses were two-sided, conducted with a significance level of 0.05 (*α* = 0.05), using R (version 4.3.2; R Foundation for Statistical Computing, Vienna, Austria).

### Determination of clustering stratification

We calculated the Calinski-Harabasz (CH) index, which measures the ratio of between-cluster to within-cluster variance, and selected the optimal number of clusters yielding the highest CH value [21, 22]. However, in high-dimensional settings (e.g., monthly scale data with 12 features), ensuring sufficient intra-cluster sample size is critical to avoid unreliable clustering outcomes. Specifically, when the sample size within a cluster is smaller than the data dimensionality, the clustering process may become unstable and prone to overfitting, capturing noise rather than meaningful patterns [23]. To address these challenges, we established a constraint on the maximum cluster number (*k*_*max*_) to guarantee that the average intra-cluster sample size exceeds the number of dimensions (*d* = 12). The optimal number of clusters within each region was then determined using the CH index, with the range of possible clusters set from 1 to *k*_*max*_. The formula for this constraint is *k*_*max*_ = ⌊*N*/*d*⌋. Applying this to China's 340 prefecture-level cities (*N* = 340) with a feature dimensionality of *d* = 12, the maximum cluster number *k*_*max*_ is determined by the ratio *k*_*max*_ = ⌊*N*/*d*⌋ = ⌊340/12⌋ = 28. If further hierarchical clustering is needed, the same formula can be applied iteratively to determine the maximum number of sub-clusters at each subsequent level, ensuring methodological consistency. We then used Spatial Kluster Analysis by Tree Edge Removal (SKATER) with first-order edge adjacency of spatial polygons as the constraint to stratify the data based on the optimal number of clusters. SKATER is a spatially constrained clustering algorithm that partitions spatial data into homogeneous groups, considering both similar attribute values and spatial contiguity, by incrementally removing edges from a minimum spanning tree [24].

### Determination of the sample size

After determining the number of strata, the sample size for each stratum was calculated using the Neyman method [25], and simple random sampling was then conducted within each stratum 100 times, with overall sample sizes ranging from 20 to 320 in increments of 10. We plotted a curve of average root mean square error (RMSE) against sample size, where the RMSE was calculated from the difference between the predicted incidence rates, obtained through Ordinary Kriging interpolation with a Spherical semivariogram at unselected cities, and the actual values. The absolute value of the derivative was calculated based on the change in RMSE with varying sample sizes, representing the sensitivity of RMSE improvement to changes in sample size. We selected the sample size corresponding to an absolute derivative value of 1, as adding one more city beyond this point would only reduce the RMSE by less than 1/100,000. This implies that further increases in the sample size would no longer significantly improve accuracy [26, 27].

### Selection of representative sample cities

We performed 100 samplings based on the sample size determined in the previous step and selected the plan with the minimum RMSE from them. Subsequently, we applied cyclic optimization to finalize the monitoring network. The specific process is as follows: (i) We calculated the Spearman's rank correlation between the true and predicted weekly incidence rates for each city. (ii) Cities with non-significant correlations (*P* > 0.05) were identified. These cities were poorly predicted by the existing monitoring network and thus needed to be added to enhance monitoring effectiveness. (iii) After incorporating these poorly predicted cities into the monitoring network, we utilized the updated monitoring network to perform Kriging interpolation and generate new predicted incidence rates. (iv) Based on these updated predictions, we recalculated the Spearman's rank correlation for all cities. This process was repeated iteratively. In each iteration, new cities with non-significant correlations were identified and added to the network. The stopping criterion was met when all cities exhibited a significant correlation (*P* < 0.05) between their true and predicted weekly incidence rates.

### Evaluation of spatial and temporal representativeness

For overall evaluation, the RMSE and Spearman's rank correlation coefficient were used to assess the mean bias and correlation between the predicted and true annual incidence rates across all cities.

For spatial feature evaluation, the consistency of global Moran's *I* between the predicted and true values was assessed. The consistency of hotspot regions was evaluated using the sensitivity, specificity, and accuracy indices for the predicted local Getis-Ord G* values, with the true values serving as the gold standard [28].

For temporal feature evaluation, Spearman's rank correlation coefficient was used to assess the correlation between the predicted and true weekly incidence rates for each city. The annual percent change (APC), average annual percent change (AAPC), joinpoints, and their 95% confidence intervals (*CI*) from Joinpoint regression were compared between the true and predicted values. The comparison focused on whether the number of joinpoints was consistent and whether the 95% *CI* for the predicted APC, AAPC, and locations of joinpoints included the corresponding true values. The consistency in the number and location of joinpoints reflects the ability to identify critical time points for changes in disease information. Consistency in APC reflects the ability to identify short-term trend changes, while consistency in AAPC evaluates the ability to capture the long-term trends in directions and magnitudes [29].

### Comparison of SCSS with the conventional methods

The conventional methods included *K*-means, traditional stratified sampling, and simple random sampling. The number of clusters and sample sizes of these conventional methods were the same as those used in the SCSS method. The *K*-means stratified sampling method included stratifying based on the results of *K*-means clustering, followed by simple random sampling within each stratum. Traditional stratified sampling divided the samples into eastern, central, and western regions, according to the standards of the National Bureau of Statistics [30]. Within each region, the samples were further stratified into high, medium, and low categories based on incidence rates. We compared the distribution of RMSE across SCSS, *K*-means, traditional stratified sampling, and simple random sampling over 100 iterations. A lower RMSE signifies more accurate predictions, while a more centralized RMSE indicates greater stability of the method.

## Results

### Results of the monitoring network design

**Fig. 2:**
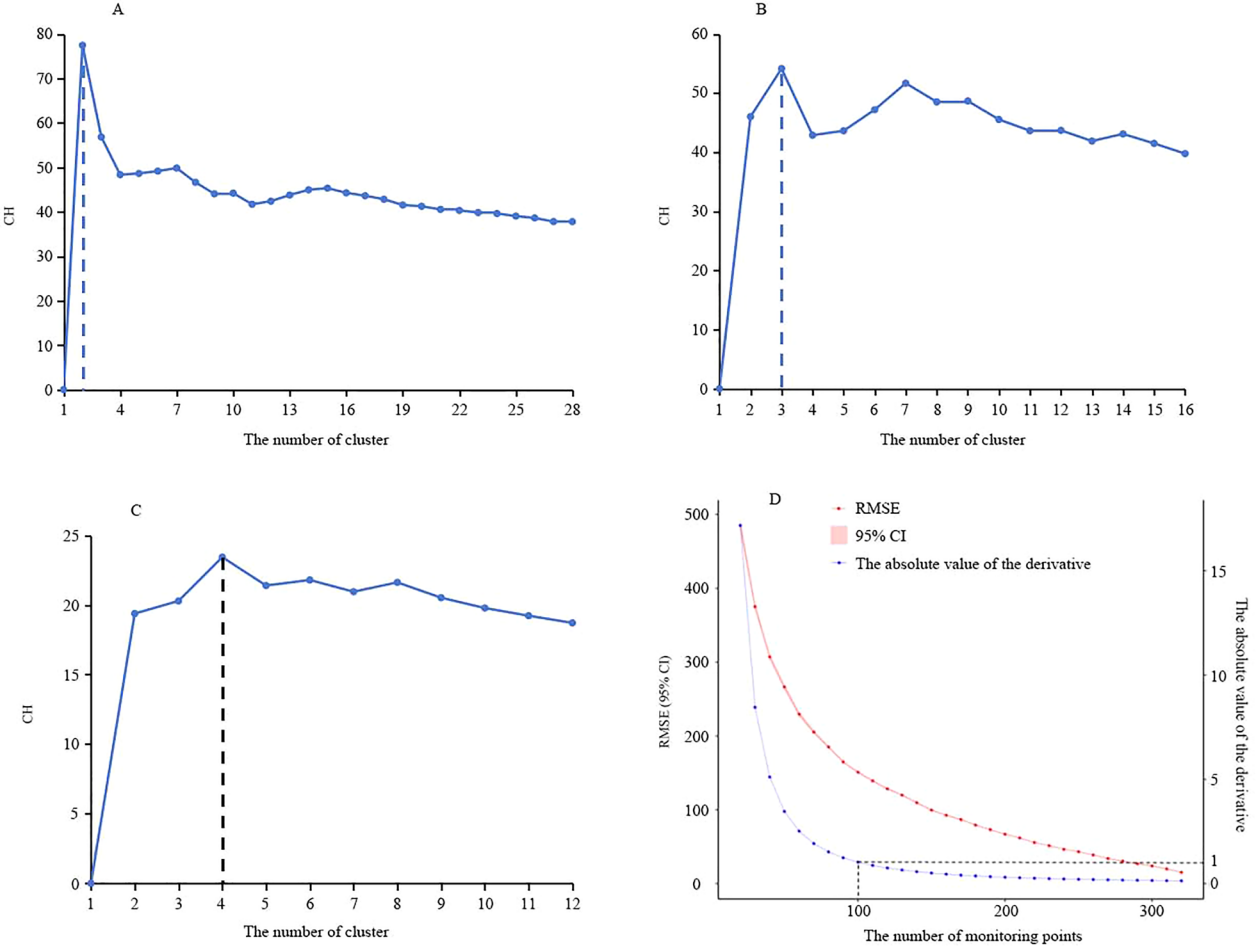
Design process of the monitoring network based on Spatial Cluster Stratified Sampling (SCSS). **A**, **B**, **C** Variation of Calinski-Harabasz (CH) values under different cluster numbers for the national, northern region, and southern region; **D** variation of Root mean square error (RMSE), its 95% confidence interval (*CI*), and the absolute value of the derivative with increasing sample size, with a narrower 95% *CI*

**Fig. 3:**
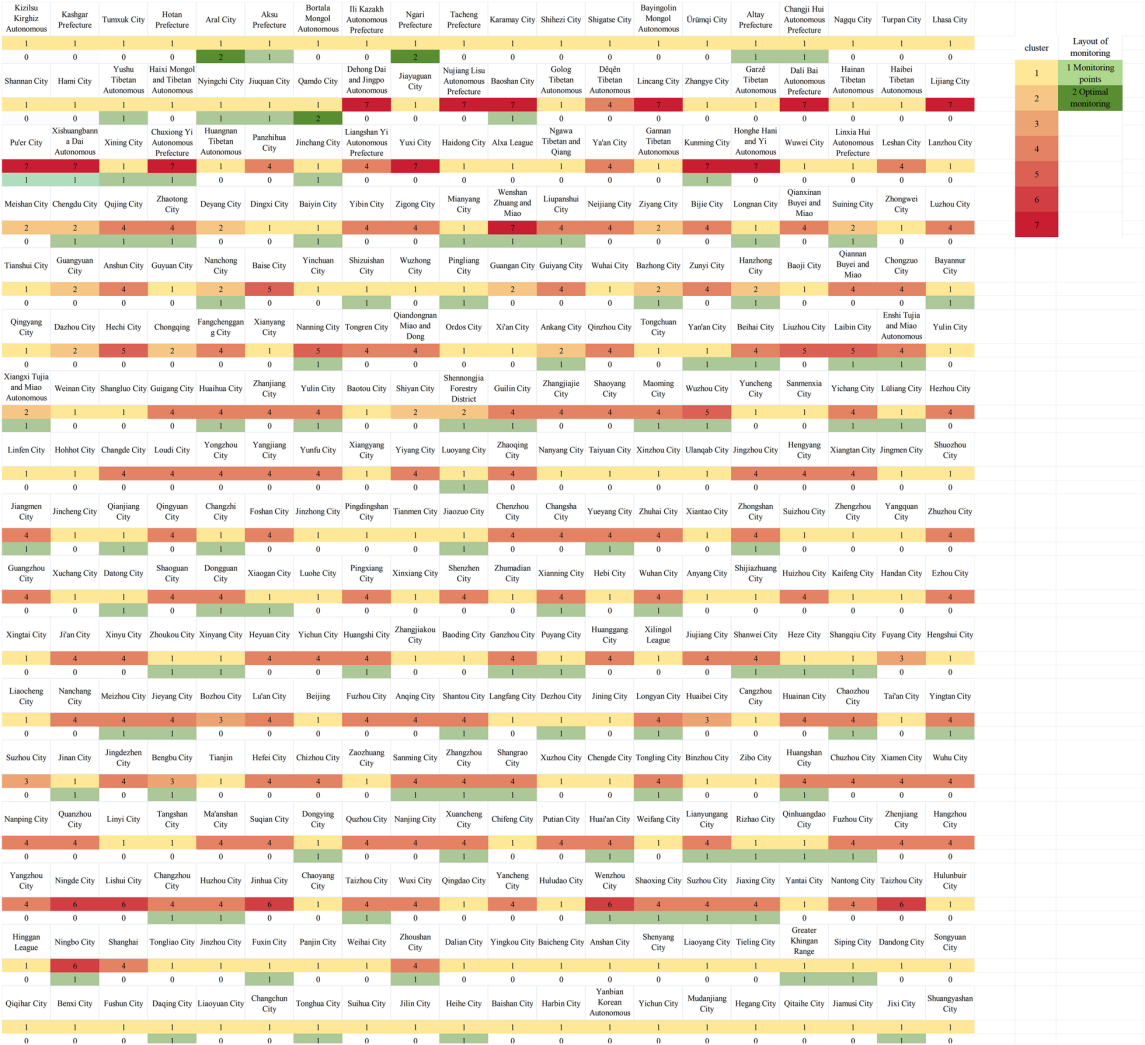
Clustering results and the selected representative sample cities

The initial clustering analysis, utilizing the CH index across a range of 1 to 28, identified two clusters, thereby segmenting the dataset into northern and southern regions (Fig. 2A). Further analysis within the northern region, comprising 196 cities, established a *k*_*max*_ of 16 (*k*_*max*_ = ⌊196/12⌋) and a CH index range of 1–16. This subsequent clustering within the northern region yielded three optimal clusters. Similarly, the southern region, with 144 cities, had a *k*_*max*_ of 12 (*k*_*max*_ = ⌊144/12⌋) and a CH index range of 1–12, and further clustering within the southern region resulted in four optimal clusters. Consequently, the final total number of clusters was seven (Fig. 2B, C). The sample size was initially set at 100, balancing cost and benefit (Fig. 2D). The three cities—Qamdo and Ngari in the Tibet Autonomous Region, and Aral in the Xinjiang Uygur Autonomous Region—which showed no significant correlation (*P* > 0.05) between the actual and predicted weekly incidence rates were added to the plan, resulting in a total of 103 cities (Fig. 3).

### Evaluation of the representativeness of the monitoring network

A significant correlation was observed between the predicted and true annual incidence rates across all cities, with Spearman's rank correlation coefficient *r* = 0.81 (*P* < 0.001) and an RMSE of 134 per 100,000.

**Fig. 4:**
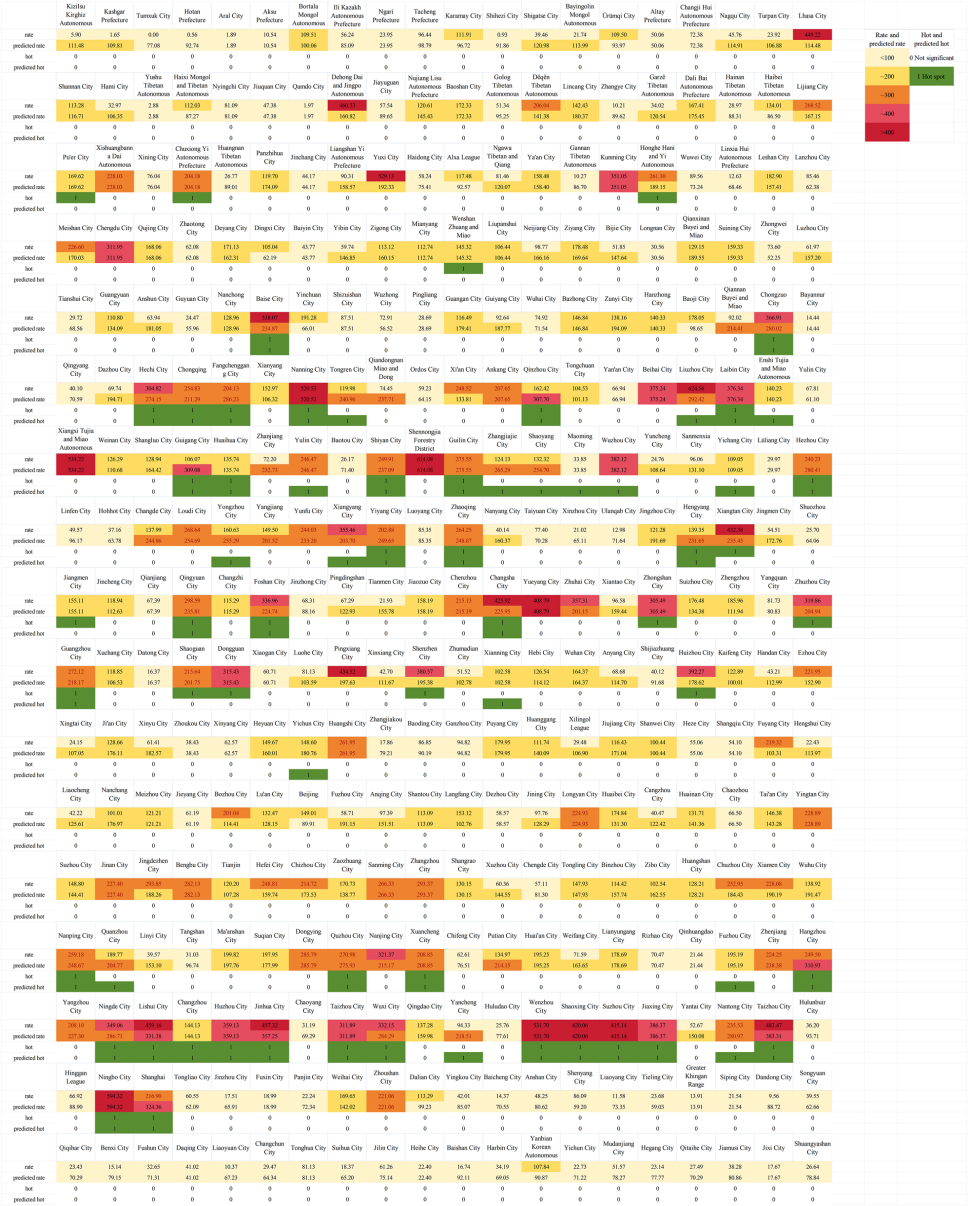
Spatial distribution and local Getis-Ord G* analysis of annual incidence. In the figure, “rate” indicates the true HFMD incidence rate in 2018 and “predicted rate” stands for the predicted one. Similarly, “hot” represents the hotspot of true HFMD incidence rate in 2018 and “predicted hot” represents that of the predicted value

The spatial distribution of the predicted values is shown in Fig. 4, which closely resembles the spatial distribution of the true values in Fig. 4. The predicted values exhibited a positive spatial correlation (Moran's *I* = 0.66, *P* = 0.001), which aligned well with the true values (Moran's *I* = 0.57, *P* = 0.001). The local spatial autocorrelation of the predicted values is depicted in Fig. 4, where the distribution of hotspot areas closely matches the true scenario (Fig. 4). The sensitivity, specificity, and accuracy were 0.76, 0.91, and 0.87, respectively.

**Fig. 5:**
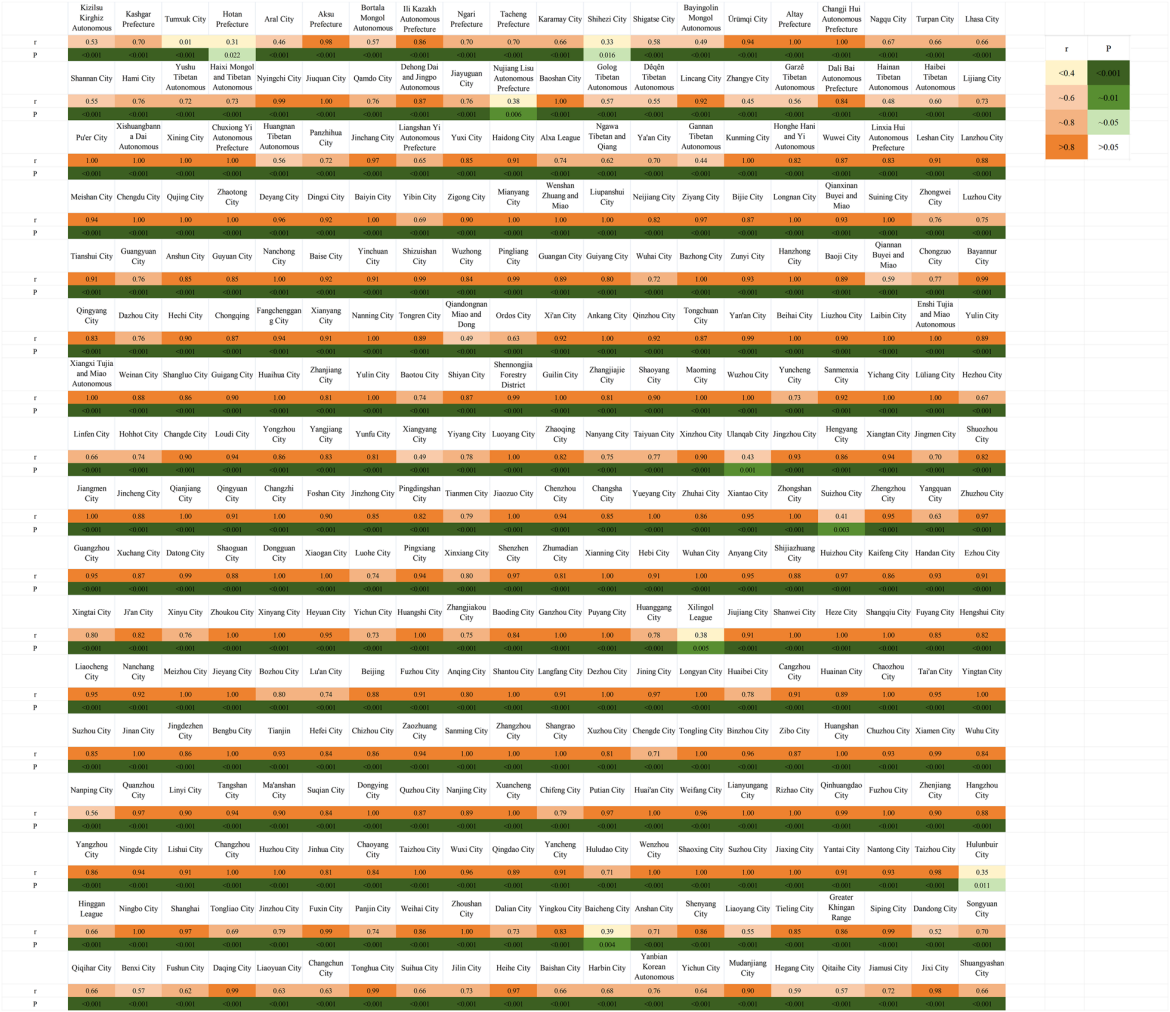
Evaluation of temporal representativeness of the monitoring network. In the figure, “r” indicates correlation coefficients of true and predicted weekly HFMD incidence rates for each city; “P” indicates significance of true and predicted weekly HFMD incidence rates for each city

**Fig. 6:**
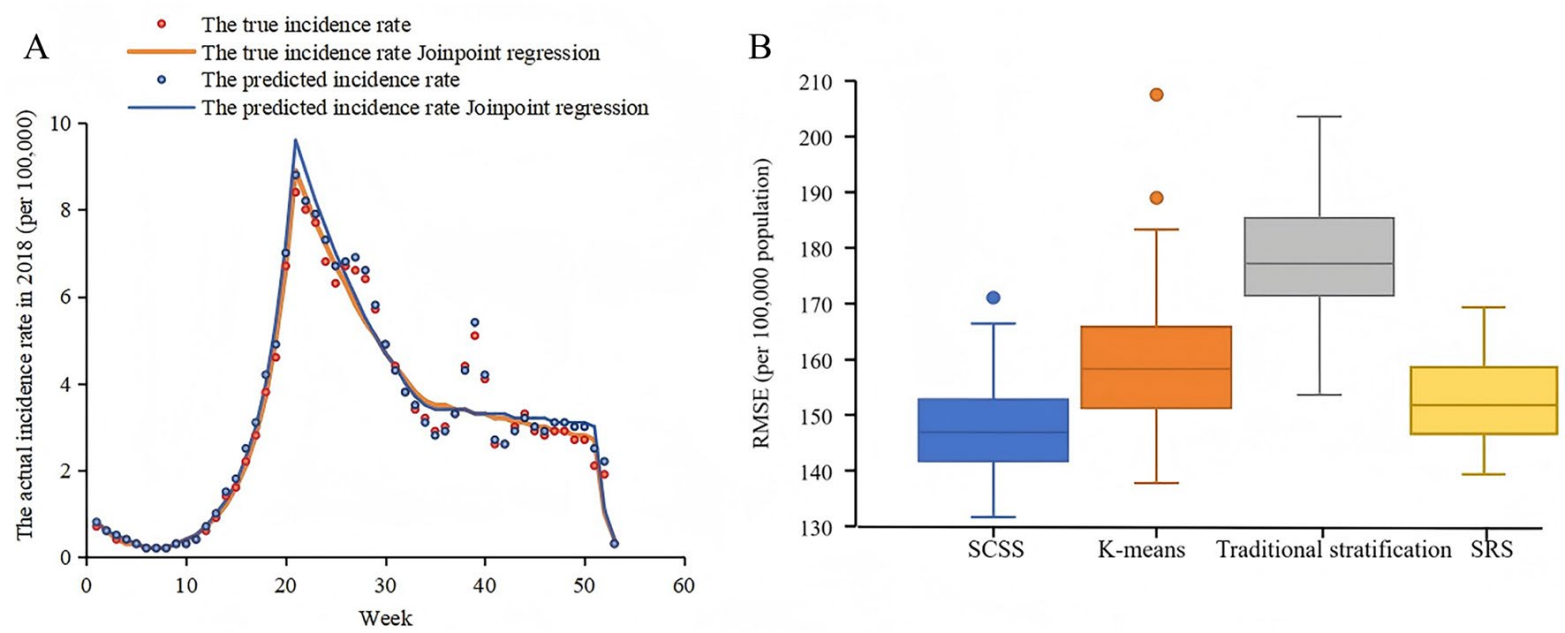
Joinpoint regression analysis and effectiveness of different methods. **A** Joinpoint regression analysis of true and predicted HFMD incidence rates; **B** Boxplots of the RMSE for different methods. In the figure, RMSE stands for Root Mean Square Error, SCSS stands for Spatial Cluster Stratified Sampling, and SRS stands for Simple Random Sampling

There were significant correlations between the predicted and true weekly incidence rates for all cities (*P* ≤ 0.05). 70% of cities showed *r* > 0.8, with 48% of these cities having *r* > 0.9 (Fig. 5). The number of joinpoints in the predicted values matched those of the true values, and the 95% *CI* of the predicted joinpoint locations, APC and AAPC contained the true values (Fig. 6A and Table 1).

**Table 1:** Joinpoints, APC, AAPC, and their 95% confidence intervals (*CI*) for true and predicted values

Indicator	Joinpoint regression for true incidence rate		Joinpoint regression for predicted incidence rate	
	Value	95% *CI*	Value	95% *CI*
Joinpoints	7	6 to 8	7	6 to 8
	21	20 to 22	21	20 to 22
	34	25 to 43	34	29 to 40
	51	50 to 51	51	50 to 51
APC(%)	− 23.17	− 28.47 to − 16.70	− 23.18	− 28.61 to − 16.71
	33.49	30.54 to 36.83	33.53	30.73 to 36.88
	− 6.76	− 13.40 to − 4.21	− 7.56	− 12.96 to -5.29
	− 1.55	− 3.08 to 2.34	− 0.77	− 2.25 to 1.95
	− 63.17	− 68.82 to − 50.71	− 62.28	− 68.10 to − 48.19
AAPC(%)	− 1.35	− 2.01 to − 0.62	− 1.22	− 1.89 to − 0.43

*APC* Annual percent change, *AAPC* Average annual percent change, *CI* confidence intervals

### Comparison of SCSS with three other methods

The RMSE medians (first quartile, third quartile) for SCSS, *K*-means, traditional stratified sampling, and simple random sampling were 147 (142–153), 158 (151–166), 177 (171–185), and 152 (147–159), respectively. The RMSE of SCSS was the lowest and most centralized, the RMSE of *K*-means was relatively low but more dispersed, and the RMSE of traditional stratified sampling was the highest and most dispersed (Fig. 6B).

The monitoring network was validated with the HFMD data in 2019 and demonstrated robust and effective performance, with strong representativeness across overall, spatial, and temporal dimensions (see Additional file 1).

## Discussion

SCSS was first proposed in this study to establish an infectious disease monitoring network. This approach takes spatial clustering of disease into account and is flexible enough to adopt new techniques for spatial clustering analysis, with this study using the SKATER as an example. SCSS was found to have greater accuracy and stability compared to traditional methods that did not consider spatial factors. The representative sample cities selected by the SCSS approach comprehensively reflect and represent the national HFMD epidemic, as assessed using RMSE, correlation, Moran's *I*, hotspot analysis, and Joinpoint regression from a spatiotemporal perspective. The SCSS approach selected 103 cities from a total of 340, reducing the sample size by 237, resulting in a 70% reduction in monitoring costs. These findings highlight the potential value of the SCSS approach in significantly enhancing the representativeness and reliability of future infectious disease monitoring networks. Its application bears important public health implications, such as more accurate estimation of disease burden, more efficient allocation of health resources, and earlier identification of emerging hotspots.

A well-designed monitoring network is essential for reducing monitoring costs and improving efficiency. It involves two key aspects: determining the sample size and selecting the representative sample cities. Selecting the appropriate sample size is crucial, as too large a sample size may lead to excessive costs, while too few can result in poor quality. Unlike existing studies, which often set the sample size based on covering a target population fraction, our approach uses mathematical derivatives to quantify the diminishing trend of marginal benefits. It explores the dynamic relationship between cost and benefit and identifies a critical threshold. At this threshold, where the marginal return of additional resource input falls below expectations, selecting this point as the optimal sample size ensures efficient resource allocation and avoids unnecessary waste. When selecting representative sample cities, a well-planned setting scheme maximizes coverage, preventing blind spots and redundant monitoring, thereby improving efficiency. Traditional stratified random sampling does not fully account for attribute values when creating strata and neglects spatial information, leading to inadequate representativeness within the strata. In contrast, our approach, SCSS, uses the SKATER clustering method, treating each cluster as a separate stratum and performing random sampling within each stratum. This approach accounts for both attribute values and spatial adjacency, ensuring high homogeneity within the strata and identifying representative sample cities. This study employed SCSS to address both of these issues in designing the monitoring network, achieving an overall RMSE of 134 per 100,000. To achieve the same RMSE, the *K*-means, traditional stratified sampling, and simple random sampling methods required 122, 140, and 120 cities, respectively, representing increases of 19, 33, and 17 cities compared to SCSS. Therefore, using design methods that consider spatial autocorrelation, such as SCSS, is beneficial for selecting representative sample cities.

This study compared the SCSS approach with three conventional methods, revealing significant differences in sampling accuracy and stability. Regarding sampling accuracy, traditional stratified sampling uses a fixed stratification approach based on geographic regions and incidence rates, but these geographic divisions may not align with the actual distribution of disease data, making this approach less effective. In contrast, the *K*-means method clusters the data based on the similarity of attribute values (e.g., incidence rates), ensuring the homogeneity within each stratum; however, it does not account for the spatial structure of disease data. The SCSS method, on the other hand, considers both attribute values homogeneity and spatial adjacency, further improving within-stratum homogeneity. As a result, SCSS provides the most accurate predictions. Regarding sampling stability, traditional stratified sampling may result in considerable attribute differences within each stratum due to the insufficient use of attribute data when defining strata. As a result, random sampling within each stratum across different iterations may select samples with notable attribute differences, affecting the stability of the sampling process. In each iteration of the *K*-means algorithm, the k initial centroids are randomly selected, and the variability in their positions can influence the final clustering results, thereby introducing instability into the outcomes [31]; SCSS, however, is not influenced by the choice of initial centroids, thus avoiding the fluctuations in clustering results caused by differing initial conditions. As a result, SCSS generates a unique clustering scheme once the number of clusters is determined, producing the most stable results [32].

Despite the potential advantages shown by SCSS, there are still several issues that warrant further exploration. First, different time scales in SCSS may result in varying clustering outcomes. A fine scale may create overly strict cluster boundaries, causing samples that should belong to the same group to be assigned to different clusters. This can lead to clusters that represent only small, localized features of the data, and such outcomes may lack practical significance. In contrast, a coarse scale may overlook important variations and subtleties. Therefore, achieving an appropriate scale is crucial for ensuring accurate analysis. We compared clustering results at weekly and monthly scales and selected the monthly scale, as it provided an appropriate number of clusters and a more balanced distribution of samples across different clusters. Future research should further explore the optimal clustering scale to maximize data utilization and optimize clustering results. However, at the present time, there are no standard methods to be utilized. Second, while the SCSS method provides a general sampling framework, its generalizability to diseases with different spatial patterns or transmission dynamics requires further validation in future studies. Third, although our study demonstrates that the selected static monitoring points can maintain representativeness for several years, adapting to changing epidemiology over longer time periods would necessitate the development of methods for dynamic optimization of the monitoring network. Lastly, due to the use of aggregated city-level data and reported case data, potential ecological fallacy and reporting bias are unavoidable.

## Conclusions

Our study developed a new method, called SCSS, to establish monitoring networks for future infectious diseases. Our results demonstrated the advantages of SCSS in handling the data of infectious diseases, that often exhibit spatial autocorrelation. The monitoring network showed strong spatiotemporal representativeness. This study provides a valuable methodological reference for designing future infectious disease monitoring networks, with the potential to significantly reduce monitoring costs and improve efficiency.

## Supplementary Information

Additional file 1.

## Data Availability

Relevant data are available from the corresponding author on request.

## Abbreviations

SCSS: Spatial cluster stratified sampling
HFMD: Hand, foot, and mouth disease
SKATER: Spatial Kluster analysis by tree edge removal
RMSE: Root mean square error
95% *CI*: 95% confidence intervals
APC: Annual percent change
AAPC: Average annual percent change
CH: Calinski-Harabasz
