# Supplementary material for: A spatial clustering-based approach to design monitoring networks of infectious diseases: a case study of hand, foot, and mouth disease
Source: Infect Dis Poverty. 2025 Jul 28;14:76. doi: 10.1186/s40249-025-01331-7 (PMC12302676; doi:10.1186/s40249-025-01331-7)
Supplement: Supplementary file 1 — Additional file 1. [file 40249_2025_1331_MOESM1_ESM.docx]

**A Spatial clustering-based approach to design the monitoring points of infectious diseases: a** **case study of hand, foot, and mouth disease**

Shuting Li^[[1]](#footnote-0)†^, Yuanhua Liu¹^†^, Ke Li^2†^, Zengliang Wang^3^, Michael P. Ward^4^, Wei Tu^5^, Jiayao Xu¹, Rui Yuan¹, Lele Zhang^2^, Na Wang¹, Jidan Zhang¹, Yu Zhao¹, Henry S Lynn¹, Zhaorui Chang^6^*, Zhijie Zhang^1,2^*

**Additional file 1**

TABLE OF CONTENTS

[SUPPLEMENTARY DEFINITIONS 1](#_Toc199256455)

[SUPPLEMENTARY FIGURES 2](#_Toc199256456)

[Figure S1: 2](#_Toc199256457)

[Figure S2: 3](#_Toc199256458)

[Figure S3: 4](#_Toc199256459)

[Figure S4: 5](#_Toc199256460)

[SUPPLEMENTARY TABLES 5](#_Toc199256461)

[Table S1: 5](#_Toc199256462)

# SUPPLEMENTARY DEFINITIONS

The hand, foot and mouth disease (HFMD) clinically diagnosed case is defined as a patient presenting with the papular or vesicular rash on the hands, feet, mouth, or buttocks, with or without fever. The typical rash presents as macules, papules, and vesicular. The rash is surrounded by an inflamed red halo, there is less fluid in the vesicular, it is not painful or itchy, and the rash recovers without crusting or scarring. Atypical rashes are usually small, thick, hard, and few, and sometimes petechiae and ecchymoses are visible. Certain types of enteroviruses, such as Coxsackie virus A6 and Coxsackie virus A10, can cause severe skin lesions, with the rash appearing as large vesicular, accompanied by pain and itching, and may not be confined to the hands, feet, or mouth.

The HFMD laboratory-confirmed case is defined as a case with laboratory evidence of enterovirus infection, including enterovirus A71 (EV-A71), coxsackievirus A16 (CV-A16), or other non-EV-A71 and non-CV-A16 enteroviruses, detected by reverse transcription polymerase chain reaction (RT-PCR), real-time RT-PCR, or viral isolation.

# SUPPLEMENTARY FIGURES


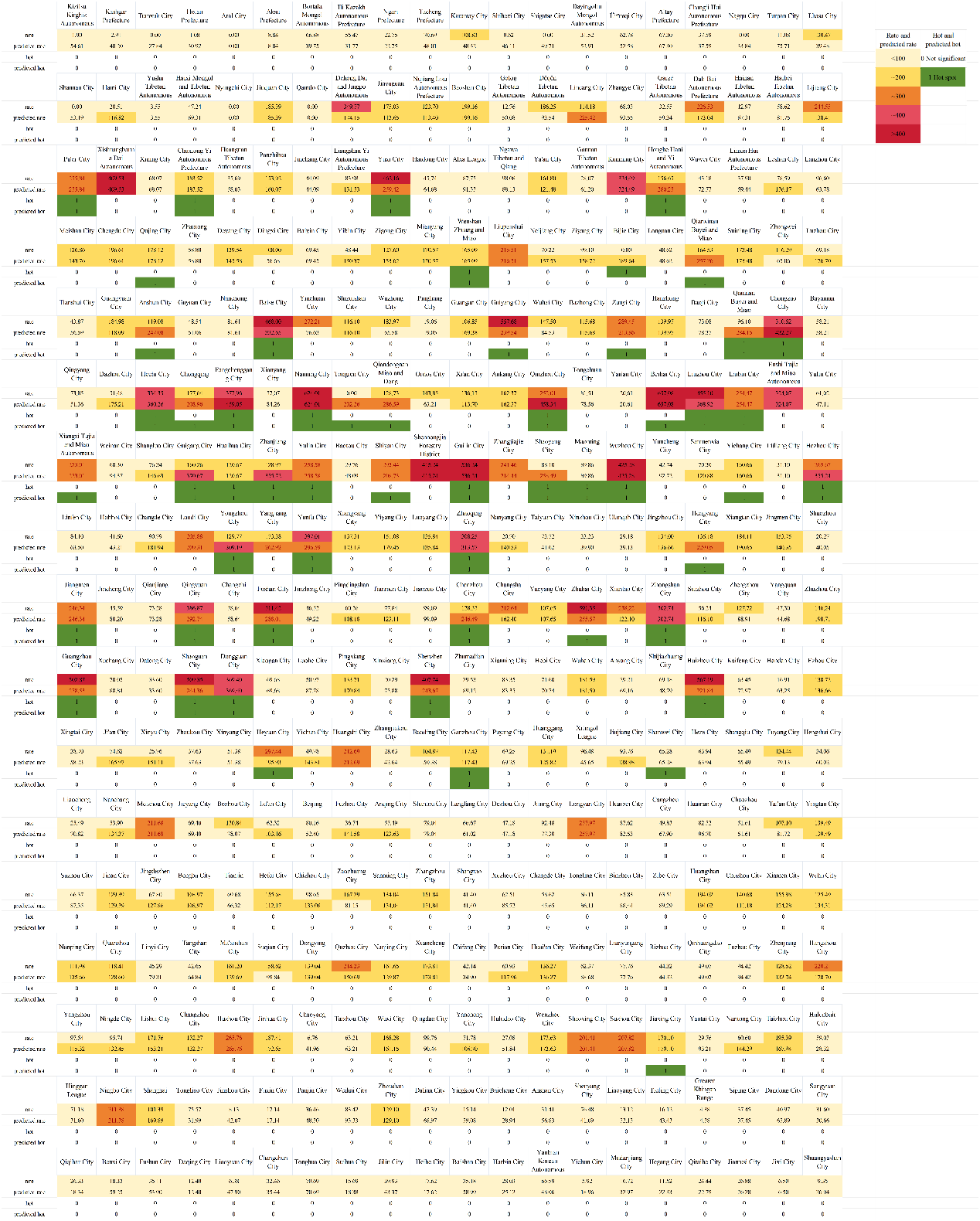


Figure S1: spatial distribution and local Getis-Ord G* analysis of annual incidence. In the figure, “rate” indicates the true HFMD incidence rate in 2019 and “predicted rate” stands for the predicted one. Similarly, “hot” represents the hotspot of true HFMD incidence rate in 2019 and “predicted hot” represents that of the predicted value


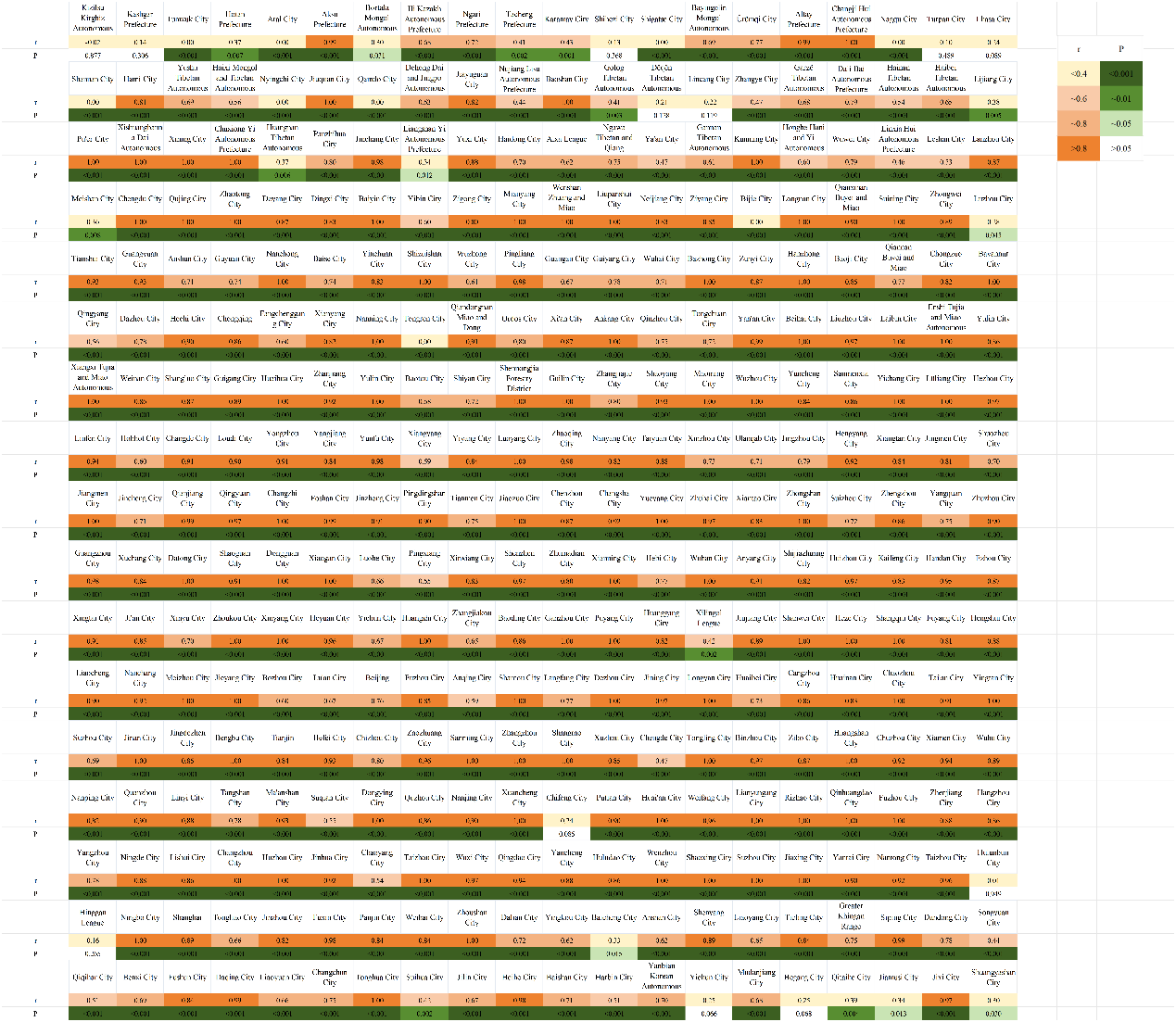


Figure S2: evaluation of temporal representativeness of the monitoring network in 2019. In the figure, “r” indicates correlation coefficients of true and predicted weekly HFMD incidence rates for each city; “P” indicates significance of true and predicted weekly HFMD incidence rates for each city


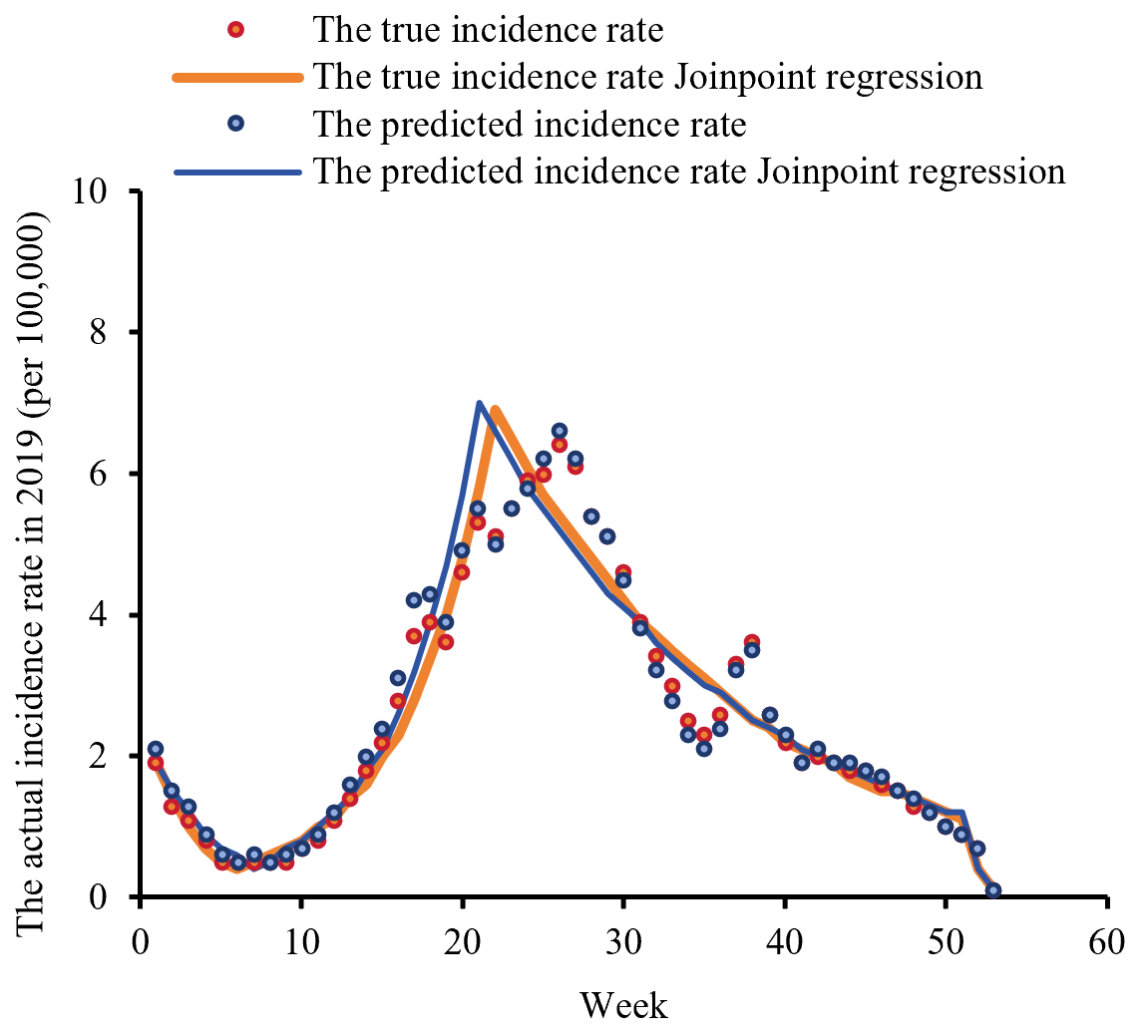


Figure S3: Joinpoint Regression Analysis of True and Predicted HFMD Incidence Rates in 2019


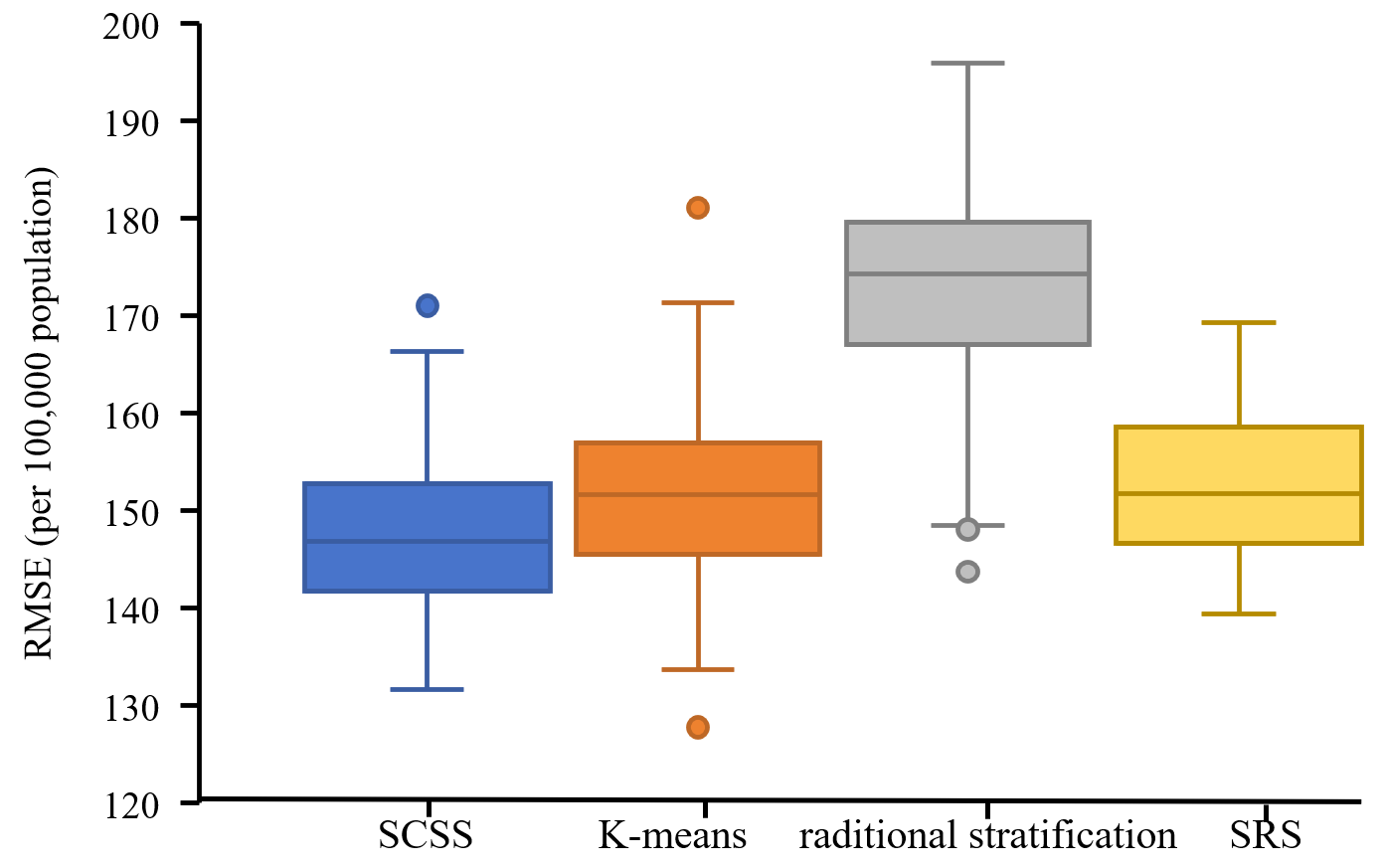


Figure S4: Boxplots of the RMSE for Different Sampling Methods in 2019

# SUPPLEMENTARY TABLES

Table S1: Joinpoint, APC, AAPC, and Their 95% Confidence Intervals for True and Predicted Values in 2019

| Indicator | Joinpoint regression for true incidence rate | | Joinpoint regression for predicted incidence rate | |
| --- | --- | --- | --- | --- |
|  | Value | 95%CI | Value | 95%CI |
| Joinpoints | 6 | 5 to 7 | 7 | 6 to 8 |
|  | 22 | 21 to 23 | 21 | 20 to 22 |
|  | 51 | 50 to 51 | 51 | 50 to 51 |
| APC（%） | -27.27 | -38.01 to -18.58 | -21.85 | -30.95 to -14.51 |
|  | 19.75 | 17.20 to 22.86 | 21.77 | 18.38 to 26.19 |
|  | -6.06 | -6.87 to -5.26 | -5.76 | -6.58 to -4.93 |
|  | -66.72 | -72.33 to -51.73 | -66.78 | -72.92 to -50.86 |
| AAPC（%） | -5.10 | -5.96 to -4.25 | -5.07 | -5.96 to -4.20 |

1. ^†^Shuting Li, Yuanhua Liu, Ke Li are Co-first authors.

   *Corresponding author:

   Zhijie Zhang

   E-mail address: [epistat@gmail.com](mailto:epistat@gmail.com)

   Zhaorui Chang

   E-mail address: [changzr@chinacdc.cn](mailto:changzr@chinacdc.cn) [↑](#footnote-ref-0)
